# Super-resolved quantum ghost imaging

**DOI:** 10.1038/s41598-022-14648-2

**Published:** 2022-06-20

**Authors:** Chané Moodley, Andrew Forbes

**Affiliations:** School of Physics, University of the Witwatersrand, Johannesburg, 2000 South Africa

**Keywords:** Single photons and quantum effects, Imaging and sensing

## Abstract

Quantum ghost imaging offers many advantages over classical imaging, including low photon fluxes and non-degenerate object and image wavelengths for imaging light sensitive structures, but suffers from slow image reconstruction speeds. Image reconstruction times depend on the resolution of the required image which scale quadratically with the image resolution. Here, we propose a super-resolved imaging approach based on neural networks where we reconstruct a low resolution image, which we denoise and super-resolve to a high resolution image. To test the approach, we implemented both a generative adversarial network as well as a super-resolving autoencoder in conjunction with an experimental quantum ghost imaging setup, demonstrating its efficacy across a range of object and imaging projective mask types. We achieved super-resolving enhancement of $4\times$ the measured resolution with a fidelity close to 90$\%$ at an acquisition time of N$^2$ measurements, required for a complete N $\times$ N pixel image solution. This significant resolution enhancement is a step closer to a common ghost imaging goal, to reconstruct images with the highest resolution and the shortest possible acquisition time.

## Introduction

Ghost imaging is an alternative image acquisition technique which utilises the correlations between two spatially separated fields of light to reconstruct an image of an object, therefore, photons that have not physically interacted with the object are used^1,2^. Individually each field cannot offer any image information on the object, however the correlations between them allows for the reconstruction of an image^3,4^. Ghost imaging was originally demonstrated as a quantum entanglement phenomenon^5^ and as a result of a spontaneous parametric downconversion (SPDC) process^6^, and recently shown with entanglement swapped photons^7^ and with symmetry engineered quantum states^8^. It was later shown that classical correlations can similarly be used in a ghost imaging experiment^9–11^. Quantum ghost imaging was initially thought to produce higher resolution images, however, it has been shown that in both quantum and classical cases images of almost identical quality are produced^2,12^. Advantageously, the use of quantum light allows for imaging at low light levels, demonstrating a higher signal to noise ratio and visibility^12,13^. Particularly, quantum ghost imaging is useful for biological imaging applications where it is beneficial to reduce the risk of photo-damage to light sensitive matter^14^.

A common ghost imaging goal is to reconstruct images with the highest resolution and the shortest acquisition times^15^, however, a limitation that ghost imaging faces is the inefficient imaging speed. Imaging speeds depend on the number of measurements needed to reconstruct the image which scales quadratically with the required resolution^16^. This imposes a practical limit on applications requiring high resolution images. Earlier raster scanning implementations were used^5^, which evolved to more timely methods using a single-pixel bucket detector and pre-computed binary intensity fluctuation patterns^17^, single-pixel scanning methods^18,19^ and Fourier single-pixel scanning methods^20^. The imaging speed remained unsatisfactory and so too did the number of measurements required to reconstruct the image^21,22^. Attempts to improve and enhance image quality and resolution focused on employing a pseudo-inverse ghost imaging technique via a sparsity constraint^23^, employing a Schmidt decomposition for image enhancement^24^, and imaging based on Fourier spectrum acquisition^25^. Deep-learning has recently gained a lot of interest due to robust enhancement and denoising capabilities in image resolution. Neural networks have been used in under-sampling the object and employing neural networks to enhance the reconstructed image^26,27^, performing Poisson noise reduction and using neural networks for image upscaling^28^, using neural networks to denoise the image^29^, using autoencoders as a self-supervised approach to enhance image quality^30^, and super-resolution imaging in the far-field with neural networks^31^.

In this work we outline a novel approach to producing super-resolved images in a quantum ghost imaging experiment. We experimentally reconstruct a low resolution image, which we denoise, and super-resolve without losing the finer details of the image. A lossless high resolution super-resolved image is achieved with fewer measurements. After implementing super-resolving neural networks we trained the networks to super-resolve images acquired from a complete ghost image reconstruction, i.e. N$^2$ measurements where N $\times$ N pixels is the image resolution. The N $\times$ N pixel image is then super-resolved to a 4N $\times$ 4N pixel image. Additionally, we implemented a reconstruction algorithm to suppress noise levels by leveraging noise-suppression characteristics from three different reconstruction algorithms. We start by describing our image reconstruction method, followed by our neural network description and experimental details, and finally present our results, where we demonstrate significant super-resolving capabilities. We believe that this novel approach to obtaining lossless super-resolved images will prove valuable to both quantum and classical ghost imaging experiments focused on obtaining high resolution images.

## Image reconstruction methods

**Figure 1:**
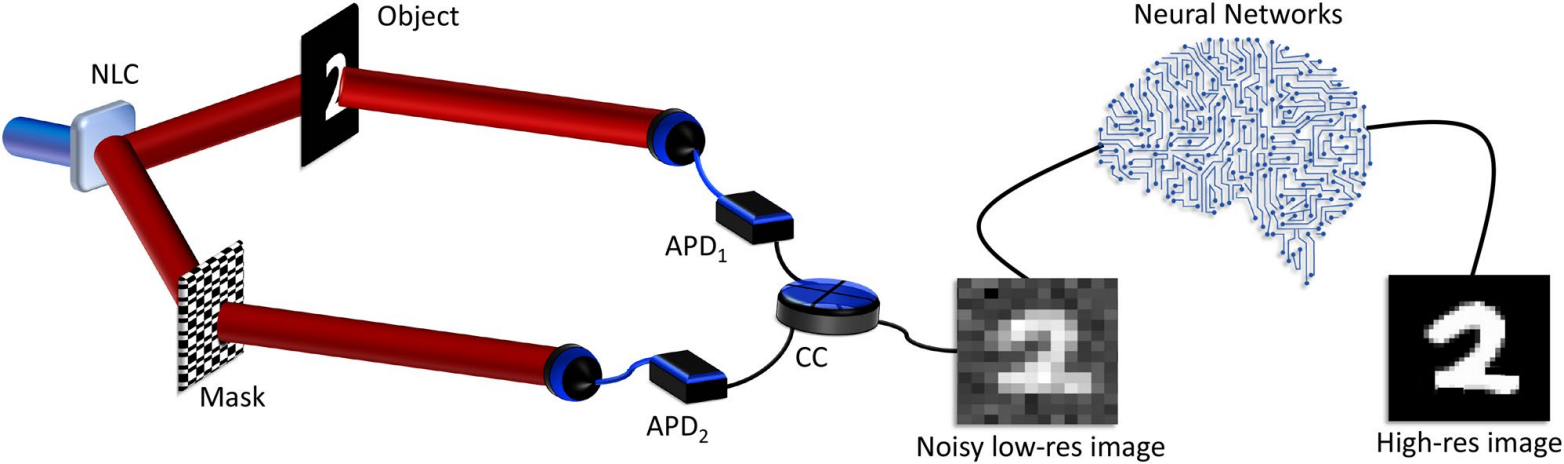
Conceptual sketch of our all-digital ghost imaging optical setup. Entangled photons are spatially separated along two arms. One photon interacts with the object and is collected by a bucket detector. The other photon is collected by a spatially resolving detector comprising a patterned mask and a bucket detector. Each detector is connected to a coincidence counting (CC) device to perform coincidence measurements. The reconstructed low res image is then sent through a series of neural networks for denoising and super-resolving to a higher resolution.

Figure 1 conceptually illustrates our all-digital super-resolved ghost imaging (GI) concept. The photon correlations necessary for ghost imaging, in our work, arise from a quantum source^5,6,32,33^. The required position correlations arise from the spatial entanglement of the signal and idler photons produced by a spontaneous parametric downconversion (SPDC) process using a non-linear crystal (NLC)^34^. Entangled photon pairs are spatially separated into two independent paths, one to illuminate the object (object arm) and one which is collected by a spatially resolving detector (reference arm). Our spatially resolving detection system is realised by displaying a series of binary patterns (masks) on a spatial light modulator (SLM), the projection is then collected by a bucket detector. The measured correlations then provide information on the similarity (or overlap) between the object and each mask. Scanning through a series of masks is a lengthy process which scales with the required resolution and is a great area of interest to the ghost imaging community^26–28,33,35,36^. Importantly, the patterns must form a complete basis to completely reconstruct the image. Once we have scanned through a series of masks and have reconstructed the image at a low resolution (low res), we then exploit artificial intelligence algorithms to denoise and super-resolve the image. This process produces super-resolved high resolution (high res) images without needing to measure at a higher resolution. Super-resolving neural networks allow us to measure at a low resolution while achieving up to 4$\times$ super-resolving resolution enhancement.

Traditionally the image is reconstructed as a linear combination of all masks weighted by the coincidences and is expressed as the traditional second order ghost imaging (TGI) reconstruction algorithm^21^:

1 $$\begin{aligned} I = \sum _{i=1}^{N} c_{i} \mathbf{M} _{i}, \end{aligned}$$

where *I* is the image, $c_{i}$ the coincidence counts, and $M_{i}$ the mask for the $i{th}$ measurement. We refer to $c_{i}$ as the coincidence counts for ease of understanding as we weight the masks by the respective coincidence counts to recontruct the image, however $c_{i}$ is the overlap between the object and mask - which is proportional to the coincidence counts. The TGI reconstruction brings forth a large background which typically suppresses the signal, the reconstructed image is, therefore, of low quality. To improve this, many studies calculate the joint probability distribution applied to imaging to improve the signal to noise ratio^37–40^. Using the joint probability distribution as a reconstruction algorithm requires that the accidental counts be subtracted from the coincidences so as to reduce the noise. We term this method accidental subtracted ghost imaging (ASGI). The image is reconstructed as follows:

2 $$\begin{aligned} I = \sum _{i=1}^{N} (c_{i} - a_{i}) \mathbf{M} _{i}, \end{aligned}$$

where $a_{i}$ is the accidental counts for the $i{th}$ measurement. Typical to ghost imaging image reconstruction, in a further effort to increase the signal to noise ratio, is the removal of the DC component, this removes the background effect and greatly improves the reconstructed image quality (TGIDC):^16,41–44^

3 $$\begin{aligned} I = \sum _{i=1}^{N} (c_{i} - \bar{c}_{N}) \mathbf{M} _{i}, \end{aligned}$$

where $\bar{c}_{N}$ is the ensemble average of the coincidences over *N* measurements. Leveraging noise suppression characteristics from the above reconstruction algorithms, we propose a novel reconstruction algorithm which merges the above and suppresses the background effect in a more efficient manner. The reconstruction takes into account both the accidentals and the removal of the DC component as follows:

4 $$\begin{aligned} I = \sum _{i=1}^{N} ((c_{i} - \bar{c}_{N}) - (a_{i} - \bar{a}_{N})) \mathbf{M} _{i}, \end{aligned}$$

where $\bar{a}_{N}$ is the ensemble average of the accidental counts over *N* measurements. We have termed this accidental subtracted ghost imaging with DC term removal (ASGIDC). We show in the experimental section that this reconstruction algorithm performs better than other commonly used algorithms by using their noise suppression characteristics in combination, producing the greatest object-image fidelity. The reconstructed image, utilising the ASGIDC algorithm, was clearer than the other images, demonstrating the highest signal-to-noise ratio and a largely suppressed background.

## Super-resolving network details

**Figure 2:**
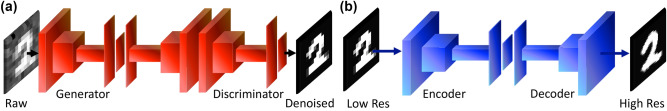
Schematic diagrams of the implemented neural network architectures showing the series of implemented 2-dimensional convolutional layers. (**a**) Schematic diagram of the implemented generative adversarial network (GAN), trained on simulated data to denoise the reconstructed image. (**b**) Schematic diagram of the implemented super-resolving autoencoder (SR AutoE), trained to enhance the resolution of an image.

Our super-resolving neural network architectures were designed and implemented to enhance the image resolution without resizing the image. Resizing an image to a higher resolution is lossy i.e. fine details are lost. While experimentally reconstructing a higher resolution image in a ghost imaging experiment leads to exponentially long image reconstruction times. In a 2-fold manner, by utilising neural networks, we can reconstruct at a low resolution, denoise and super-resolve the image to a high resolution. This yields efficient measurement times while the output is enhanced up to 4$\times$ the measured resolution. Importantly we note that throughout the paper our use of the word “resolution” is with reference to the pixels per inch (PPI) resolution description^45^. Our networks are split into two distinct parts. Part one involves generating images from simulated data that are indistinguishable from real data, in application real experimental data was sent into this network for denoising. Part two consists of a super-resolving network to enhance the image resolution and to resolve fine details without any loss occurring, the images generated in part one are then passed through this network for resolution enhancement. The implemented neural network process is shown schematically in Fig. 2, from left to right: the simulated ghost image, from the MNIST dataset, goes through two network architectures - one to denoise and one to enhance the resolution of the image. The neural networks are optimised to the MNIST digit dataset, prior knowledge of the object type is important when selecting a training dataset. For a binary object not of the digit category one would need to select a training dataset corresponding to the category of the object, i.e. for alphabets, birds, or cells we would utilise a training dataset whose characteristics are similar to that of the object of interest.

We implemented a generative adversarial network (GAN) as shown in Fig. 2a. The idea around GANs is that there exists two models that work coherently: a generative model and a discriminative model. The generator was given the task of generating or creating images that look natural or similar to the original data. The discriminative model was tasked with determining whether or not these images looked natural or were created artificially. A GAN is often referred to as a zero-sum game. The generator tries to fool the discriminator while the discriminator tries to not get fooled by the generator. As both models trained through alternating optimization, both methods were improved until a point where the generated data was indistinguishable from the MNIST digit dataset. The models were then allowed to generate synthetic data with similar statistical properties to the point where the synthetic data and the real data were indistinguishable. In our case the generator was tasked to take in the simulated data (data generated from simulating the ghost imaging experiment) and to then generate images that were indistinguishable from the MNIST digit data. Image indistinguishability is calculated by minimising the loss function when training the model. When the loss function reaches a plateau at a minimum, training is considered complete and the images are indistinguishable with respect to their statistical moments. The aim was to train the model to generate real data and to finally recognise the experimental data as real after it has been denoised. When building the training set we used the MNIST digit dataset, each image within the dataset was used as a digital object and the ghost imaging image reconstruction was simulated by the use of random masks. Our ghost imaging simulation was implemented similarly to that in Ref.^44^ and as described by Eq. (4). Finally we added random noise drawn from a normal distribution to further simulate what the experimental results would look like. The result of an image from the simulated dataset is shown on the left of Fig. 2a. The synthetic dataset is therefore matched to the experimental realisation by simulating what the experimental realisation would look like based on utilising the MNIST digit dataset as objects in a ghost imaging experiment. The generator is a convolutional autoencoder that contains both an encoder and a decoder. We used a series of 2D convolutional and transpose convolutional layers in the network architecture, these layers are connected by max pooling and up sampling layers respectively. The final layer of the generator uses the $\tanh$ activation function as this layer outputs the denoised image. The discriminator, in general, is used to classify an image as real or fake. The discriminator is a convolutional classifier and was used only to validate the generator’s predictions. The final layer of the discriminator is a dense layer with a sigmoid activation for classification, a schematic diagram is shown in Fig. 2a. Our implemented GAN is similar to that implemented in Ref.^46^ with the caveat of an entirely different training set. When using the GAN to denoise experimental data we only utilised the generator.

For the second network we implemented a super-resolving autoencoder (SR AutoE) architecture This is a specialised neural network due to its super-resolving capabilities, further details on the architecture are contained in Refs.^47–49^. Autoencoders are used in applications where image denoising, image reconstruction from sparse sampling, and data compression and decompression are important. These neural networks are used as a dimensionality reduction technique in principle component analysis and are used in super-resolving applications to generate high resolution images. Autoencoders are a type of neural network which compress the input into a latent-space representation. The output is then reconstructed from the latent-space representation, thereby making autoencoders a self-supervised, machine learning algorithm. Part two of our network architecture required less augmentation of the training set. We used the GAN output at 12 $\times$ 12 pixels and the MNIST dataset scaled to 32 $\times$ 32 pixels to train the SR AutoE, the architecture of which is shown in Fig. 2b. Our SR AutoE consists of a 2D convolutional autoencoder architecture similar to that which appears in the GAN. For the encoder, 2D convolutional layers are separated by max pooling layers, while the decoder consists of transpose convolutional layers separated by up sampling layers. The schematic architecture of our SR AutoE is shown in Fig. 2b.

A limitation present in all networks is that their capacity to generalise decreases when given data that was not previously seen. In the case of both networks, the GAN and SR AutoE, the signals from the experimental data are generalised to the features that each individual network learnt during training.

## Experimental details

**Figure 3:**
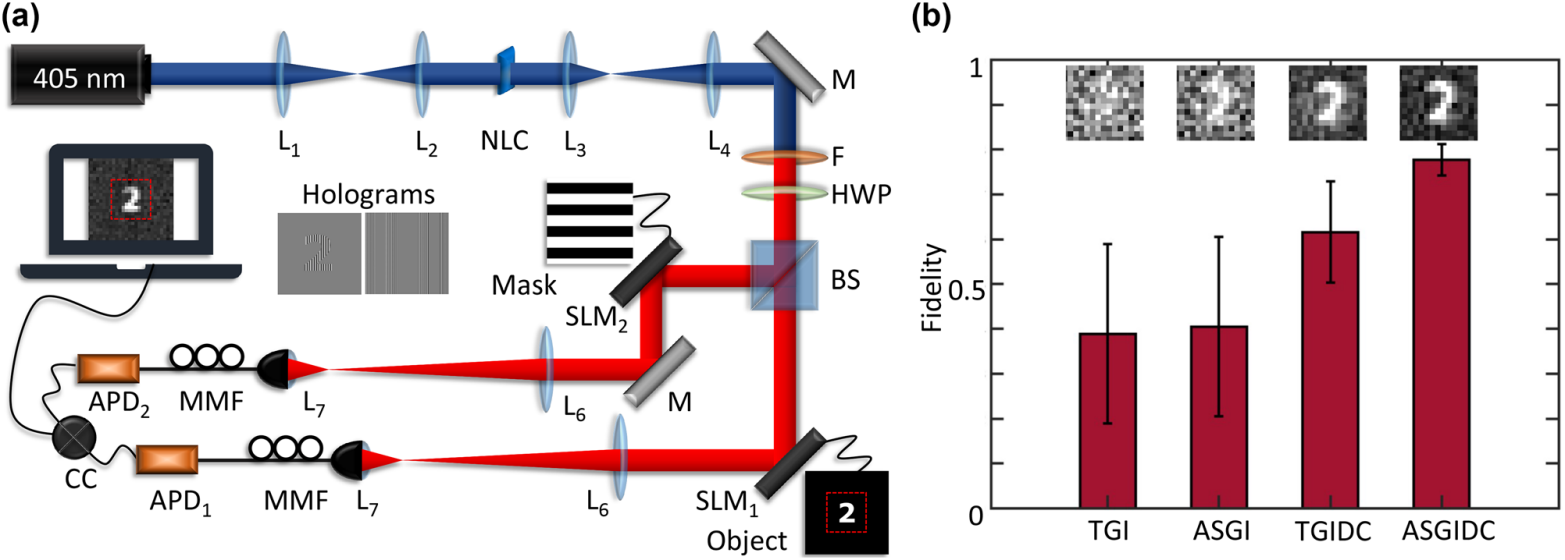
Schematic diagram of the implemented quantum ghost imaging setup followed by results from the tested reconstruction algorithms. (**a**) Entangled bi-photons are produced via SPDC at the NLC. The entangled photons are spatially separated by a 50:50 BS and imaged onto a SLM in each path. Coincidence measurements are done between both paths and the coincidences are used to reconstruct an image of the object. (**b**) Fidelity results of the different reconstruction algorithms, insets show the image after a complete reconstruction.

Figure 3a shows a schematic sketch of the quantum ghost imaging optical setup implemented for this work. Light from an OBIX 405LX diode laser at a wavelength of $\lambda = 405$ nm was used to pump a temperature controlled type 1 Periodically Poled Potassium Titanyl Phosphate (PPKTP) NLC. The optimal temperature was set to obtain co-linear emission of entangled bi-photons at wavelengths of 810 nm each via SPDC. Any unconverted pump photons were filtered out by means of a THORLABS bandpass filter (F) centered at a wavelength of $\lambda$ = 810 nm (FWHM = 10 nm). Vertically polarized down-converted photons emitted from the NLC were then rotated to horizontal polarization by a half-waveplate (HWP). The polarization rotation was done to ensure optimal modulation of the impinging photons by a HOLOEYE PLUTO-2.1 spatial light modulator (SLM) situated in each arm. The entangled photon beam was separated into two paths by a 50:50 beam splitter (BS) and each arm was then directed to a SLM where the required holograms were displayed. The SLM in one arm displayed the digital object to be imaged while the SLM in the other arm was used to display the required scanning masks. A blazed grating was added to both holograms to separate the first diffracted order from the zeroth, unmodulated, order. Photons from the first diffracted order in each arm were then coupled into THORLABS multimode optical fibres (MMFs), of core size 62.5 $\mu$m, connected to Perkin Elmer - SPCM-13 avalanche photo-diodes (APDs) for photon detection. Coincidences were measured within a 25ns window by our National Instruments (NI) photon counting device (CC). The crystal, SLMs and fibre entrances were placed at conjugate planes. In the experimental setup any background or ambient light contributed to noise, resulting in a noisy image reconstruction. Additionally shot noise contributed to noisy images. Shot noise occurs due to the discrete nature of electric charge, therefore our coincidence counter as well as our APDs contributed to shot noise.

Prior to starting our ghost imaging measurements we routinely test for, and confirm the presence of entanglement in controlled superposition states of orbital angular momentum (OAM). Specific to this work we tested for the entangled nature of OAM states generated by SPDC through the violation of a suitable Clauser-Horne-Shimony-Holt (CHSH) Bell inequality^50^. Our experiment was carried out similarly to that reported in Ref.^51^. By using appropriate holograms displayed on the SLMs in each arm, we can generate two-photon states entangled within a two-dimensional OAM subspace. For these test measurements we coupled the photons from the first diffracted order into THORLABS single-mode fibres, with a core size of 5 $\mu$m. We found a Bell parameter of $S = 2.71 \pm 0.07$ for the $l = \pm 1$ subspace therefore successfully violating the Bell inequality. To confirm entanglement we also performed a full quantum state tomography (QST) for the $l = \pm 1, p = 0$ subspace in the Laguerre-Gaussian basis and report a fidelity of $F = 0.962$, a concurrence of $C = 0.927$, and a linear entropy of $S_L = 0.061$. Additionally, our quantum contrast was $Q = 20.18 \pm 0.44$.

For our ghost imaging experiments we used binary sets of Hadamard and random masks as detailed in some of our previous work^16,44^. Random patterns, since they are not correlated to each other, form an over-complete set with randomly distributed binary pixels, each with a 50$\%$ probability of being either on or off. Our Hadamard masks are generated by extracting the Walsh functions from the native MATLAB function for Hadamard matrices where *N* is the order of the Hadamard matrix. By performing the outer product between columns of a Hadamard matrix of order *N*, the result is a complete set of $N^2$ Hadamard masks of $N \times N$ pixel resolution. Choosing the order of the Hadamard matrix will therefore result in the corresponding mask resolution. The mask resolution determines the resolution at which the object will be imaged. A higher resolution results in a larger number of basis elements and therefore an increased number of masks is needed to reconstruct the image. Increasing the resolution has direct consequences on the reconstruction time, the number of Hadamard masks required to form a complete set scales as $N^2$ and so there is a trade-off between reconstruction time and image resolution. A 32 $\times$ 32 pixel image would, therefore, require 1024 masks. The random masks form an over-complete set and we therefore used approximately twice the amount of random masks, here we used 2000. The Hadamard and random masks were generated in a pre-measurement step in MATLAB. LabVIEW was used to control the SLMs, and to record the signal emitted from the APDs and received from the coincidence counting device. LabVIEW was also used to perform the real-time image reconstruction. We took measurements for two different objects (digits 2 and 3) using three common reconstruction algorithms as well as our own reconstruction algorithm. We used 32 $\times$ 32 pixel Hadamard and random masks to image the object, however due to a large black border around the main area of the object (as shown in Fig. 3a) the effective resolution of the reconstruction is a 12 $\times$ 12 pixel image (shown as insets in Fig. 3b). The images were reconstructed using four different algorithms so as to test and confirm that our algorithm performed the best. The results of which are shown in Fig. 3b. The insets show the image after a complete set of reconstructions for each algorithm and we used the object-image fidelity as a test of image quality. The object-image fidelity is a fidelity measure calculated from the normalised mean squared error, the correlation, the contrast, and the luminance between the two images (in this case the digital object and the image) and is based on the quality index factor proposed in Ref.^52^ and detailed in Ref.^44^. We saved the final image after reconstructing with the total number of masks and focused only the effective resolution area indicated by the dotted red lines in Fig. 3a. For the effective resolution, we selected the area of the image that only contained the reconstructed object and a shorter black border, i.e. 12 $\times$ 12 pixels. Figure 3b shows that Eq. (4) performed better than the other commonly used reconstruction algorithms. The image was then sent into both neural networks for denoising and super-resolving. The fidelity of the image was calculated at each step in the reconstruction, denoising, and super-resolving processes.

## Results

**Figure 4:**
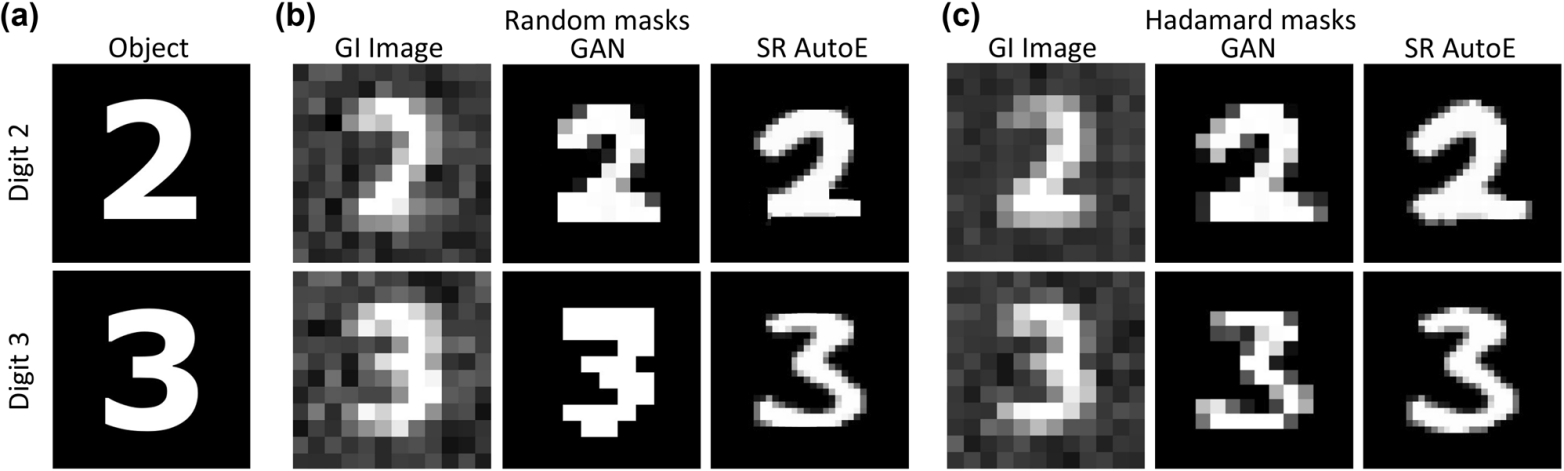
Results of the image reconstructions for both digits that were reconstructed, denoised and super-resolved. (**a**) The digital objects that were used in the experiment. (**b**) Results of the image reconstruction by random masks—from left to right: the image obtained from the experiment, the denoised image from the GAN, the super-resolved image output from the SR AutoE. (**c**) Results of the image reconstruction by Hadamard masks—from left to right: the image obtained from the experiment, the denoised image from the GAN, the super-resolved image output from the SR AutoE.

We implemented our neural networks, built the dataset, and trained the models as a pre-measurement step. It was important for us to test the models on experimental data which was completely unseen by the networks, as such the digital object used in the ghost imaging experiment was a computer generated digit and not a handwritten digit. We varied experimental parameters such as the object, mask type, and reconstruction algorithm. The algorithm in Eq. (4) reconstructed images of a better quality as shown in the object-image fidelity tests (Fig. 3b), all images were then reconstructed with Eq. (4). Figure 4a shows the digital objects that were used while Fig. 4b,c show the results of the image reconstruction (left), the denoised image (centre), and the super-resolved image (right) for the random and Hadamard mask reconstructions, respectively. As can be seen, all images shown in Fig. 4 are of the same surface area however, the PPI differs from image to image. The GAN output has a lower PPI while the SR AutoE output has a higher PPI.

The GI image is obtained after a complete image reconstruction with 2000 random masks or 1024 Hadamard masks. In the random reconstruction, we reconstruct with approximately 2$\times$ the number of Hadamard masks so as to have an over-complete set of measurements. Visibly, it can be seen that the images reconstructed with random masks are less complete in comparison to those reconstructed with the Hadamard masks (left columns of Fig. 4b,c). This artifact is taken care of by the GAN, which was trained to not only remove the noise but to fill in the parts of the image buried heavily in noise. To produce a super-resolved image of good quality, it was necessary to implement a network that would remove or suppress the noise in the GI image reconstruction. We see that for both mask types the GAN outputs a similar denoised image, owing to the signal being the same yet is hidden by different amounts of noise. The GAN output is, therefore, reconstruction invariant and was successful in predicting the correct output from both types of mask reconstructions. After denoising our images we send them into the super-resolving autoencoder (SR AutoE). As can be seen in the final columns of Fig. 4b,c the super-resolved images are not resized images of the GAN output but rather the network has learnt to fill in the fine details that are lost with resizing/resampling.

**Figure 5:**
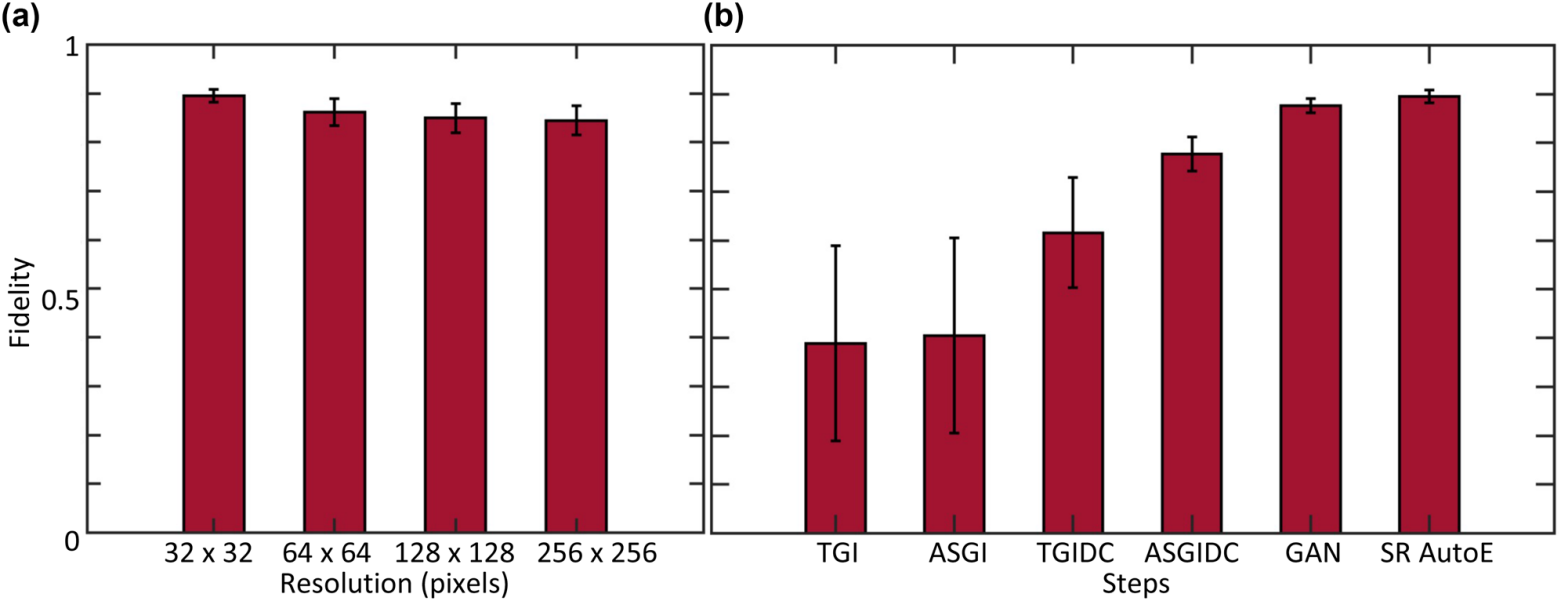
Object-image fidelity results for objects at several pixel resolutions and for each step of the process. (**a**) The results of the object-image fidelity calculated from the SR AutoE output for objects of several resolutions. (**b**) The results of the average object-image for the different reconstruction algorithms, the GAN output, and the SR AutoE output for a 32 $\times$ 32 pixel object.

To quantify the quality of the super-resolved images, we tested the object-image fidelity of objects at different resolutions with the SR AutoE output, the average results (and error bars) are shown in Fig. 5a. We denoised and super-resolved the reconstructed image, we then calculated object-image fidelity at resized object resolutions of 32 $\times$ 32, 64 $\times$ 64, 128 $\times$ 128, and 256 $\times$ 256 pixels and averaged over all the relative images, the results are shown in Fig. 5a. We show that the fidelity decreases with an increase in object resolution. However at 4$\times$ the measured resolution we have a fidelity of ~87$\%$. Thereby super-resolving to 4$\times$ the measured resolution. Comparatively, the raw images reconstructed by Eq. (4 )falls short of achieving an 80$\%$ object-image fidelity (Fig. 3b). Figure 5b displays the average object-image fidelity, over all the reconstructed images, at each step in the process. We calculated the fidelity for each of the reconstruction algorithms, we then chose the algorithm with the highest fidelity to reconstruct all our images, we sent these images into the GAN for denoising and calculated the fidelity and finally we super-resolved the image by the SR AutoE and calculated the fidelity. The fidelity increased at each step in our super-resolving process as shown in Fig. 5b. We therefore achieved super-resolved images with ~90$\%$ object-image fidelity and super-resolved to 4$\times$ the measured resolution.

## Conclusion

To summarise, we implemented a novel method to generate super-resolved images at a resolution 4$\times$ greater than that at which we measured. We designed and implemented a neural network based approach to super-resolve an image after a quantum ghost imaging image reconstruction and varied parameters such as the object and mask type. Our dataset was built from simulated data and we built, trained and optimised our networks as a pre-measurement step so as to allow for instantaneous denoising and super-resolving post-measurement. This process can be implemented in real-time and as a functional application programming interface (API) supplementary to any data acquisition software, in our case LabVIEW. We tested the performance of our new reconstruction algorithm and found that it suppressed the background with a higher signal-to-noise ratio and produces the greatest object-image fidelity. After reconstructing with our new reconstruction algorithm we sent the images into the GAN for denoising, followed by the SR AutoE for super-resolving. Visually we showed that the GAN is able to denoise the reconstructed images irrespective of the masks used for reconstruction and is therefore robust to mask types. The SR AutoE super-resolved the images closer to the MNIST dataset as this is what it was trained on. This, however, did not lead to a loss of information but rather a lossless resolution enhancement with the image super-resolving to the same digit as the object. During each step of our super-resolving process we calculated the average object-image fidelity across all image reconstructions and different objects. We showed an increase in the fidelity across all steps with the greatest fidelity achieved at the SR AutoE output. Additionally, we showed that this technique is robust to different mask types and objects. We quantified the quality of the super-resolved images at different test resolutions by calculating the object-image fidelity. By doing so we have shown 4$\times$ super-resolving capabilities with a fidelity at ~90$\%$. We believe that this is a significant step forward in producing super-resolved images from a ghost imaging reconstruction and will prove valuable to the ghost imaging community (Supplementary file).

## Supplementary Information

Supplementary Information.
