# Supplementary material for: Super-resolved quantum ghost imaging
Source: Sci Rep. 2022 Jun 20;12:10346. doi: 10.1038/s41598-022-14648-2 (PMC9209480; doi:10.1038/s41598-022-14648-2)
Supplement: Supplementary file 1 — Supplementary Information. [file 41598_2022_14648_MOESM1_ESM.pdf]

Supplementary information

Super-resolved quantum ghost imaging

Chané Moodley[1*] and Andrew Forbes[1]

[1]School of Physics, University of the Witwatersrand, Johannesburg 2000, South Africa
[*]544489@students.wits.ac.za

MATLAB code to simulate ghost imaging reconstructions

In this code we used each image of the MNIST digit dataset as a digital object in a ghost imaging simulation. We simulated what the acquired image would look like in a ghost imaging experiment, and did this for each data point in the MNIST digit dataset. We saved each simulated image after each simulated reconstruction. We used the simulated MNIST ghost image together with the original MNIST images as the dataset to train the neural network termed generative adversarial network, which is detailed below.

```
close all;
clear all;
clc;

% download the mnist.mat file from here: ...
    https://lucidar.me/en/matlab/load-mnist-database-of-handwritten-digits-in-matlab/
% save the file in the correct working directory

% load train and test images
load ('mnist.mat');

training_length = length(training.images);
test_length = length(test.images);

% Pre-allocate memory
image = zeros(32,32);
coinc = zeros(32^2,1);

% create simulated training set
for i = 1:training_length

    object = training.images(:,:,i);
    object = imresize(object, [32 32], 'nearest');

%     generate hadamard matrix for scanning masks
    h = hadamard(32);

    for j = 1:32^2
%         mask hadamard mask
        column1 = mod(i-1,n)+1;
        column2 = floor((i-1)/n)+1;
        mask = imresize(0.5 .* (h(:,column1) * h(:,column2)' +1), [32,32], 'nearest');

%         simulated overlap signal
        c = abs (sum( sum( object.*mask ) ) ).^2;
        coinc(i) = c;

        reconstruct image after each mask
        avg = mean( coinc(1:i) );
        image = image + (c-avg).*mask;

    end
```

```
    imwrite(image, ['SimulatedGhostImages/train_image_' num2str(i) '.png'], 'BitDepth', 16);

    clear image;

end


% create simulated test set
for i = 1:test_length

    object = training.images(:,:,i);
    object = imresize(object, [32 32], 'nearest');

%     generate hadamard matrix for scanning masks
    h = hadamard(32);

    for j = 1:32^2
%         mask hadamard mask
        column1 = mod(i-1,n)+1;
        column2 = floor((i-1)/n)+1;
        mask = imresize(0.5 .* (h(:,column1) * h(:,column2)' +1), [32,32], 'nearest');

%         calculate simulated overlap
        c = abs (sum( sum( object.*mask ) ) ).^2;
        coinc(i) = c;

        reconstruct image after each mask
        avg = mean( coinc(1:i) );
        image = image + (c-avg).*mask;

    end

    imwrite(image, ['SimulatedGhostImages/test_image_' num2str(i) '.png'], 'BitDepth', 16);

    clear image;

end
```

JUPYTER NOTEBOOK code for the Generative Adversarial network model

Our Generative Adversarial Network (GAN) is termed such as it is the name of a type of neural network and was implemented using the implementation model detailed in Ref. [1]. We used the simulated MNIST ghost images (called x_train) as well as the original MNIST images (called X_train) to train the network. We detail the model architecture below.

[ ]:
```
# import all the dependencies required
import numpy as np
import random
import matplotlib.pyplot as plt
%matplotlib inline
from tqdm import tqdm
from keras.datasets import mnist
from keras.layers import Input, Conv2D
from keras.layers import AveragePooling2D, BatchNormalization
from keras.layers import UpSampling2D, Flatten, Activation
from keras.models import Model, Sequential
from keras.layers.core import Dense, Dropout
from keras.layers.advanced_activations import LeakyReLU
from keras.optimizers import Adam
from keras import backend as k
```

[ ]:
```
# this is a function written to load all images within a certain repository and␣
 ↪can be
# tailored to load the images as well as the image labels. In this case we did␣
 ↪not
# need the image labels.
def load_images_to_data(image_label, image_directory, features_data, label_data):
    list_of_files = os.listdir(image_directory)
    for file in list_of_files:
        image_file_name = os.path.join(image_directory, file)
        if ".png" in image_file_name:
            img = np.array(Image.open(image_file_name).convert('L').
 ↪resize((12,12)))
            img = np.resize(img, (12,12,1))
            im2arr = np.array(img)
            im2arr = im2arr.reshape(1,12,12,1)
            features_data = np.append(features_data, im2arr, axis=0)
            label_data = np.append(label_data, [image_label], axis=0)
    return features_data, label_data
```

[ ]:
```
# load simulated ghost images using the previously defined function
x_train = np.array(Image.open('SimulatedGhostImages/train*.png').convert('L').
 ↪resize((12,12)))
x_train = x_train.reshape(1,12,12,1)
```

```
x_test = np.array(Image.open('SimulatedGhostImages/test*.png').convert('L').
 ↪resize((12,12)))
x_test = x_test.reshape(1,12,12,1)

# load the MNIST digit dataset (using X_train and X_test for the GAN)
(X_train, y_train), (X_test, y_test) =  mnist.load_data()

# the simulated ghost images together with their original MNIST counterpart were␣
 ↪used to train the GAN
# X_train - MNIST dataset
# x_train - simulated ghost images from MNIST dataset
```

[ ]:
```
# here we define the classifier architecture, we use Keras as our deep-learning
# framework and employ a series of 2 dimensional convolutional layers ending in a
# softmax which will predict category as well as output the probability of the␣
 ↪prediction

input_shape = (12,12,1)
def train_mnist(input_shape, X_train, y_train):
    model = Sequential()
    model.add(Conv2D(32, (3, 3), strides=2, padding='same',␣
 ↪input_shape=input_shape))
    model.add(Activation('relu'))
    model.add(Dropout(0.2))
    model.add(Conv2D(64, (3, 3), strides=2, padding='same'))
    model.add(Activation('relu'))
    model.add(Dropout(0.2))
    model.add(Conv2D(128, (3, 3), padding='same'))
    model.add(Activation('relu'))
    model.add(Dropout(0.2))
    model.add(Flatten())
    model.add(Dense(1024, activation = 'relu'))
    model.add(Dense(10, activation='softmax'))
    model.compile(loss = 'sparse_categorical_crossentropy', optimizer = 'adam',␣
 ↪metrics = ['accuracy'])
    model.fit(X_train, y_train, batch_size = 128, epochs = 10,␣
 ↪validation_split=0.2, verbose = 1 )
    return model
mnist_model = train_mnist(input_shape, X_train, y_train)
```

[ ]:
```
# we need to specify the GAN hyperparameters - these were fine-tuned during␣
 ↪training and testing
smooth_real = 0.8989
epochs = 200
batch_size = 128
```

```
# we used the adam optimizer as this provided us with the best results, we␣
↪tested
# various optimisers such as adagrad and adadelta as well
optimizer_g = Adam(lr=0.0002, beta_1=0.5)
# we used the adam optimizer as this provided us with the best results, we␣
↪tested
# various optimisers such as adagrad and adadelta as well
optimizer_d = Adam(lr=0.0004, beta_1=0.5)
# our input shape is of a smaller number of pixels
input_shape = (12,12,1)
```

[ ]:
```
# here we define the generator architecture - a GAN comprises a generator and␣
↪discriminator.
# We used a series of 2 dimensional convolutional layers separated by average␣
↪pooling layers,
# these layers are pre-defined in the Keras framework, we called the respective␣
↪Keras functions

def img_generator(input_shape):
    generator = Sequential()
    generator.add(Conv2D(32, (3, 3), padding='same', input_shape=input_shape)) #␣
↪32 filters
    generator.add(BatchNormalization())
    generator.add(Activation('relu'))
    generator.add(AveragePooling2D(pool_size=(2, 2))
    generator.add(Conv2D(64, (3, 3), padding='same')) # 64 filters
    generator.add(BatchNormalization())
    generator.add(Activation('relu'))
    generator.add(AveragePooling2D(pool_size=(2, 2)))
    generator.add(Conv2D(128, (3, 3), padding='same')) # 128 filters
    generator.add(BatchNormalization())
    generator.add(Activation('relu'))
    generator.add(Conv2D(128, (3, 3), padding='same')) # 128 filters
    generator.add(Activation('relu'))
    generator.add(UpSampling2D((2,2)))
    generator.add(Conv2D(64, (3, 3), padding='same')) # 64 filters
    generator.add(Activation('relu'))
    generator.add(UpSampling2D((2,2)))
    generator.add(Conv2D(1, (3, 3), activation='tanh', padding='same')) # 1␣
↪filter
    return generator

img_generator(input_shape).summary()
```

[ ]:

```
# here we define the discriminator architecture - a GAN comprises a generator␣
↪and discriminator.
# We used a series of 2 dimensional convolutional layers, these layers are␣
↪pre-defined in the Keras framework,
# we called the respective Keras functions

def img_discriminator(input_shape):
    discriminator = Sequential()
    discriminator.add(Conv2D(64, (3, 3), strides=2, padding='same',␣
↪input_shape=input_shape, activation = 'linear'))
    discriminator.add(LeakyReLU(0.2))
    discriminator.add(Dropout(0.2))
    discriminator.add(Conv2D(128, (3, 3), strides=2, padding='same', activation␣
↪= 'linear'))
    discriminator.add(LeakyReLU(0.2))
    discriminator.add(Dropout(0.2))
    discriminator.add(Conv2D(256, (3, 3), padding='same', activation = 'linear'))
    discriminator.add(LeakyReLU(0.2))
    discriminator.add(Dropout(0.2))
    discriminator.add(Flatten())
    discriminator.add(Dense(1, activation='sigmoid'))

    return discriminator

img_discriminator(input_shape).summary()
```

[ ]:
```
# here we define the entire GAN architecture which comprises a generator and␣
↪discriminator which we defined above.

def dcgan(discriminator, generator, input_shape):
    discriminator.trainable = False
    gan_input = Input(shape=input_shape)
    gen_img = generator(gan_input)
    gan_output = discriminator(gen_img)

    gan = Model(inputs=gan_input, outputs=gan_output)
    return gan
```

[ ]:
```
# we created a training function which took in all the pre-specified parameters␣
↪as well as x_train and X_train,
# we trained the GAN and plotted the generator and discriminator losses,
# once the losses reached a minimum plateau we stopped the training

def train(X_train, x_train, input_shape, smooth_real, epochs, batch_size,␣
↪optimizer_g,
          optimizer_d):
```

```
    discriminator_losses = []
    generator_losses = []
    iterations = X_train.shape[0] // batch_size
    generator = img_generator(input_shape)
    discriminator = img_discriminator(input_shape)
    discriminator.compile(loss='binary_crossentropy',optimizer=optimizer_d)
    gan = dcgan(discriminator, generator, input_shape)
    gan.compile(loss='binary_crossentropy', optimizer=optimizer_g)

    for i in range(epochs):
        print ('Epoch %d' % (i+1))
        for j in tqdm(range(1, iterations+1)):

            original = X_train[np.random.randint(0, X_train.
 ↪shape[0],size=batch_size)]
            sim = x_train[np.random.randint(0, x_train.shape[0],␣
 ↪size=batch_size)]
            generated_images = generator.predict(sim)
            dis_lab = np.zeros(2*batch_size)
            dis_train = np.concatenate([original, generated_images])
            dis_lab[:batch_size] = smooth_real
            discriminator.trainable = True
            discriminator_loss = discriminator.train_on_batch(dis_train, dis_lab)
     discriminator_losses.append(discriminator_loss)

            gen_lab = np.ones(batch_size)
            discriminator.trainable = False
            sample_indices = np.random.randint(0, X_train.shape[0],␣
 ↪size=batch_size)
            original = X_train[sample_indices]
            sim = x_train[sample_indices]
            generator_loss = gan.train_on_batch(sim, gen_lab)
            generator_losses.append(generator_loss)

            if i == 0 and j == 1:
                print('Iteration - %d', j)
                generated_images_plot(original, sim, generator)
                plot_generated_images_combined(original, sim, generator)

    return generator

generator = train(x_train, X_train, input_shape, smooth_real, epochs,␣
 ↪batch_size, optimizer_g, optimizer_d)
```

JUPYTER NOTEBOOK code for the Autoencoder network model

Here we detail the model architecture implemented for the autoencoder network which reesolves the low resolution image to a higher resolution with more pixels. In neural network terms this type of network is termed a Super-Resolving Autoencoder network.

[ ]:
```
# import all the dependencies required

from keras.datasets import mnist
import numpy as np
from keras import layers
from keras.layers import Input, Dense, Dropout
from keras.models import Model
import matplotlib.pyplot as plt
from PIL import Image
from keras.callbacks import ReduceLROnPlateau
from keras import utils
from keras.datasets import mnist
from keras import backend as K
import cv2
encoding_dim = 32
# specify number of pixels in the unsampled and upsampled images
LR = 12 # low resolution
HR = 32 # high resolution
```

[ ]:
```
# import MNIST digit dataset

(x_train, y_train), (x_test, y_test) = mnist.load_data()
```

[ ]:
```
# reshape to upsampled images

x_train = np.reshape(x_train, (len(x_train), 32, 32, 1))
x_test = np.reshape(x_test, (len(x_test), 32, 32, 1))
```

[ ]:
```
# here we created the unsampled training set from the MNIST dataset

x_train_small = []
for img in x_train:
  x_train_small.append(cv2.resize(img, dsize=(LR, LR), interpolation=cv2.
 ↪INTER_CUBIC))
x_train_small = np.asarray(x_train_small)

x_test_small = []
for img in x_test:
  x_test_small.append(cv2.resize(img, dsize=(LR, LR), interpolation=cv2.
 ↪INTER_CUBIC))
x_test_small = np.asarray(x_test_small)
```

[ ]:

```
# Here we defined the model architecture using the Keras deep-learning framework␣
↪and the pre-defined layers
# within Keras. We used a series of 2 dimensional convolutional and transpose␣
↪convolutional layers.

input_img = Input(shape=(12, 12, 1))

x = Conv2D(32, (3, 3), activation='relu')(input_img)
x = MaxPooling2D((2, 2))(x)
x = Conv2D(64, (3, 3), activation='relu')(x)
x = MaxPooling2D((2, 2))(x)
x = Conv2D(64, (3, 3), activation='relu')(x)
x = Flatten()(x)
x = Dense(49, activation='softmax')(x)

x = Reshape((7,7,1))(x)
x = Conv2DTranspose(64,(3, 3), strides=2, activation='relu', padding='same')(x)
x = BatchNormalization()(x)
x = Conv2DTranspose(64,(3, 3), strides=2, activation='relu', padding='same')(x)
x = BatchNormalization()(x)
x = Conv2DTranspose(32,(3, 3), activation='relu', padding='same')(x)
decoded = Conv2D(1, (3, 3), activation='sigmoid', padding='same')(x)

autoencoder = Model(input_img, decoded)
autoencoder.compile(optimizer='adam', loss='binary_crossentropy')
autoencoder.summary()

autoencoder.fit(x_train_small, x_train, epochs=200, batch_size=64, shuffle=True,
                validation_data=(x_test_noisy, x_test))
```

MATLAB code for generating Hadamard masks

We created Hadamard masks that were used in the experiment. These masks were read and displayed on the spatial light modulator using LabVIEW. The MALAB code is detailed below.

```
clear all;
close all;
clc;

H = 32;
V = 32;

r = 32;
h = hadamard(r);

n = r^2;

for i = 1:n

    column1 = mod(i-1,r)+1;
    column2 = floor((i-1)/r)+1;
    mask = imresize(0.5 .* (h(:,column1) * h(:,column2)' +1), [H,V], 'nearest');


%     Specify folder where to store the masks
    imwrite(mask(:, :, 1), ['HadamardMasks/mask_' num2str(i, '%05d') '.tiff']);
    disp(i)

end
```

MATLAB code for generating random masks

We created random masks that were used in the experiment. These masks were read and displayed on the spatial light modulator using LabVIEW. The MALAB code is detailed below.

```
clc;
clear all;
close all;

H = 32;
V = 32;

n = 32;

num_masks = 2*n^2;
fill = 0.5;

rng(1)

for i = 1:num_masks
    mask = imresize(fill ≤ rand(n,n),[960,960],'nearest');
    mask = double(mask);

    %     Specify folder where to store the masks
imwrite(mask(:, :, 1), ['RandomMasks/mask_' num2str(i, '%05d') '.tiff']);
    disp(i)

end
```

LabVIEW virtual instrument to control the ghost imaging experiment

In Fig. 1 we show the block diagram of the LabVIEW virtual instrument (VI) we created and used for data acquisition during our ghost imaging experiment. We show the VI architecture used during data acquisition. LabVIEW is a graphical programming language; a typical LabVIEW VI would look something like what is shown in Fig. 1. This VI allows for real-time image

reconstruction during data acquisition. The virtual instrument reads in the masks created in MATLAB and displays them on the spatial light modulator. The National Instruments (NI) coincidence counter is connected to the PC and LabVIEW is once again used to acquire the signal from the NI counter.

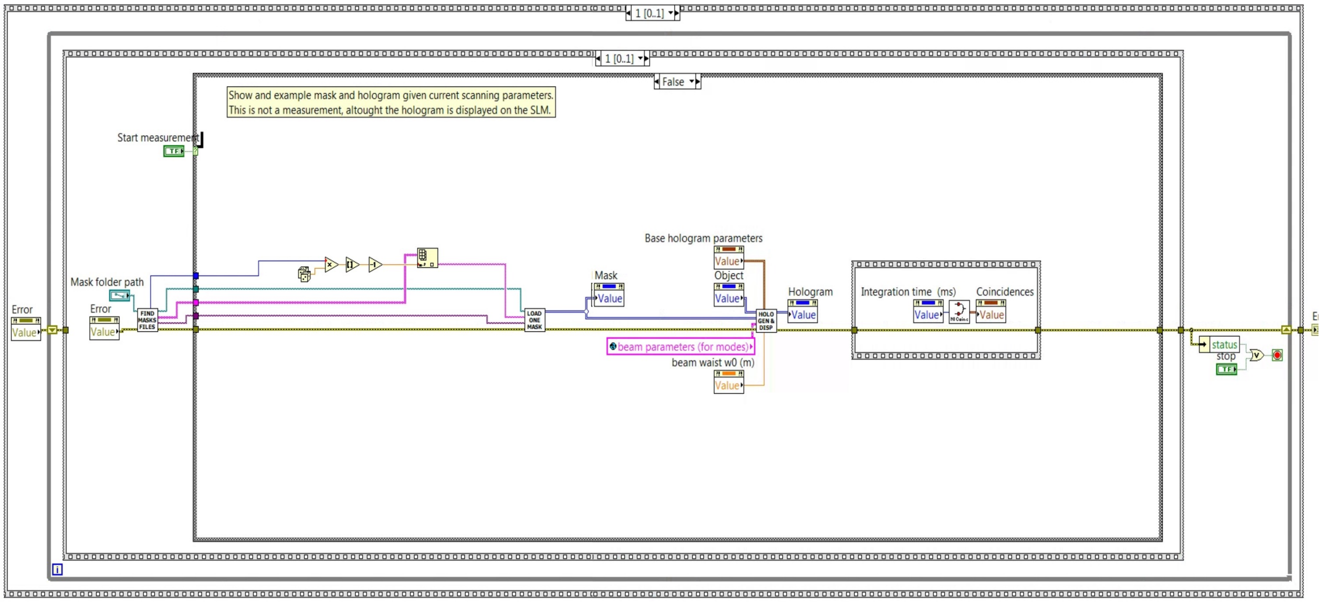

Figure 1. LabVIEW ghost imaging virtual instrument implemented for image acquisition and used to reconstruct ghost images in real-time.

References

[1] M. Lamons, R. Kumar, and A. Nagaraja, *Python Deep Learning Projects: 9 projects demystifying neural network and deep learning models for building intelligent systems*. Packt Publishing Ltd, 2018.
